# 

**Published:** 1863-02

**Authors:** 


### Editorial.

### CINCINNATI DENTAL ASSOCIATION.

It will be seen, by reference to another page, that the Dentists of Cincinnati and vicinity are meeting regularly in the capacity of a local dental association. This society suspended its meetings about one year ago, on account of the distraction every where present; it is again fully under way, with, we think, more vigor and energy than ever before. The meetings will, perhaps, be as often as once a week during the long evenings, at least we judge so from the interest at present manifested. We most cordially invite any of our professional brethren who may be, at any time of our meetings stopping in the city, to meet with us, and give us the aid of their counsel; the association will gladly welcome any such.

PERSONAL.

We would direct attention to the card of J. R. McCurdy in the advertising sheet of this number. At the time Mc went out of the dental furnishing business he placed himself in a certain condition in regard to business that was to continue for a specified time. The object of the card is to correct an impression that some seem entertain, that he has privately violated the contract; this seems to have arisen from the fact of a dental furnishing firm in Philadelphia having a McCurdy in it. But he is not our Jno. R. T.

SCALE OF PRICES

ADOPTED BY THE

### DENTISTS OF CINCINNATI AND VICINITY.

*To take effect February 1st, 1863.*

| | | | |
|---|---|---|---|
| Gold Fillings, Small and Medium Cavities, each.......... | | | $3 00 |
| " " Large and more complicated or compound........ | $4 00 | to | 10 00 |
| Restoring Crowns........ | 10 00 | to | 25 00 |
| Filling Fangs........ | 3 00 | to | 5 00 |
| Destroying Nerve........ | 1 00 | to | 3 00 |
| Separating Teeth, each Separation........ | | | 1 00 |
| Removal of Superficial Caries, each........ | 1 00 | to | 2 00 |
| Removing Tartar and Polishing Teeth........ | 1 00 | to | 5 00 |
| Extracting Teeth, each........ | 50 | to | 1 00 |
| Extra for using Battery........ | 1 00 | to | 2 00 |
| " administering Chloroform........ | 3 00 | to | 5 00 |
| Treating Alveolar Abscesses, each........ | 2 00 | to | 15 00 |
| Artificial Crowns on Roots, each........ | 5 00 | to | 12 00 |
| Full Sets of Teeth, on Gold Plate........ | 130 00 | to | 200 00 |
| " " " Rubber........ | 80 00 | to | 125 00 |
| " " Continuous Gum........ | 150 00 | to | 200 00 |
| " " Mineral Base........ | 150 00 | to | 200 00 |
| Partial Sets, from One to Four Teeth, Gold, per tooth.... | 7 00 | to | 20 00 |
| " " " Five to Ten " " " ... | 6 00 | to | 10 00 |
| " " on Rubber, 25 per cent. less than on Gold Plate. | | | |
| Artificial Teeth, on Silver Plate, half the price of Gold for same. | | | |

*The above refers to all First Operations whether regarded as Temporary or Permanent.*

For remodeling Temporary Plates, half the above price, if done within one year.

For preparing the Mouth for Inserting Artificial Teeth, $5 00 to $15 00.

For engagements not kept a fee of $2 00 may be charged unless the patient gives due notice of inability to attend.

NO GUARANTEES GIVEN.

We, the undersigned, agree to conform to the above Regulations:

J. Taft, George F. Foote,
H. R. Smith, P. Knowlton,
J. G. Cameron, W. C. Duncan,
H. A. Smith, C. H. James,
A. B. Van Osten, Samuel Wardle,
B. P. Bellknap, G. L. Hare,
B. D. Wheeler, James Taylor,
James T. Irwin, John C. Hardy,
S. L. Hamlen, W. M. Hunter.
Gam'l Jackson, D. W. Roudebush.

### CONTENTS.

### DENTAL REGISTER,

### OF THE WEST.

### Issued Monthly, at $3 a year in advance.

DEVOTED TO THE INTERESTS OF THE DENTAL PROFESSION.

**Edited by J. TAFT and GEO. WATT.**

The Volume commences in January and closes in December.

The Register being issued monthly is always fresh, therefore indispensable to the practitioner who desires to keep posted up with the new inventions and improvements which are daily developed. Neither expense nor effort will be spared to give value and variety to its contents. Articles will be illustrated with well executed engravings, whenever they will add to the interest or assist in explanation.

Its extensive circulation and frequency of issue makes it the BEST DENTAL ADVERTISING MEDIUM in the United States.

☞CONTRIBUTIONS SOLICITED.

Address Original Communications to **Geo. Watt, Xenia, O.**
Exchanges to **J. Taft**, or **Dental Register, Cincinnati, Ohio.**
Business Communications to

J. TAFT, Publisher,
56 West Fourth Street, Cincinnati, O.

JNO. T. TOLAND, Proprietor,
38 West Fourth Street, Cincinnati, O.

Communications must reach the Editor as early as the 15th, to insure insertion in the next issue.

